# Functional analysis of the *GPAT4* gene mutation predicted to affect splicing

**DOI:** 10.1080/10495398.2023.2269210

**Published:** 2023-10-31

**Authors:** Sergey A. Bursakov, Anastasia V. Kovaleva, Artyom V. Brigida, Oleg G. Zaripov

**Affiliations:** a Institution of Innovative Biotechnology in Animal Husbandry – A Branch of the Federal Research Center for Animal Husbandry Named After Academy Member L.K. Ernst, Moscow, Russia; b Federal State Budgetary Scientific Institution “All-Russia Research Institute of Agricultural Biotechnology”, Moscow, Russia

**Keywords:** *GPAT4*, alternative splicing, single nucleotide polymorphism, cattle, dairy farming

## Abstract

The *GPAT4* gene is considered as a potential functional candidate for single nucleotide polymorphism (SNP) studies in dairy cattle breeding due to its association with dairy performance in cattle by encoding an enzyme responsible for the presence of diacylglycerols and triacylglycerols in milk. Using the example of the *GPAT4* gene, we applied the minigene splicing assay to analyze the functional consequences of its variant that was predicted to affect normal splicing. The results of functional analysis revealed the sequence variations (rs442541537), transfection experiments in a wild type and mutant cell line model system demonstrated that the investigated mutation in the second intron of the *GPAT4* gene was responsible for the presence of a second exon in mature messenger RNA (mRNA). The cases of its absence in the spliced mature mRNA transcript resulted in a truncated dysfunctional protein due to the appearance of a stop codon. Thus, the discovered SNP led to alternative splicing in pre-mRNA by the 'cassette exon’ ('exon skipping’) mechanism. The studied mutation can potentially be a molecular genetic marker for alternative splicing for the *GPAT4* gene and, therefore contributes to economic benefits in cattle breeding programs.

## Introduction

Glycerol-3-phosphate 1-O-acyltransferase (EC number: 2.3.1.15) (GPAT4; AGPAT6; glycerol-3-phosphate acyltransferase 4) is a crucial enzyme for the biosynthesis of glycerolipids and triacylglycerol in eukaryotes, as well as for the conversion lysophosphatidic acid to phosphatidic acid: acyl-CoA + sn-glycerol 3-phosphate < => CoA + 1-acyl-sn-glycerol 3- phosphate. AGPAT6 is one of the 6 members in the 1-acylglycerol-3-phosphate-O-acyltransferase (AGPATs) family in *Bos taurus* with each corresponding independent gene.^1^^,^^2^ The most abundant isoform^3^ of bovine mammary gland AGPAT6 possess four conserved lysophosphatidic acid acyltransferase motifs (approximately 100 amino acids^4^) which are important for binding substrates and catalyzing the acyltransferase reactions.^5^ They are located on the COOH-terminal side of the second transmembrane domain.^6^

AGPAT6 has been recognized as microsomal glycerol-3-phosphate acyltransferase (GPAT) and renamed as GPAT4.^4^^,^^7^ Corresponding *GPAT4* gene is located on 27-th autosome of Bos taurus and incorporating 14 exons.^8^ This gene is widely expressed and can be detected at the mRNA level in most tissues examined.^5^^,^^7^^,^^9^ The microsomal *GPAT4* gene is located exclusively in the endoplasmic reticulum^10^ and is highly expressed in the mammary gland epithelium and upregulated during lactation as shown in mice and bovine.^3^^,^^9^^,^^11^

The fat content of milk determines the quality of milk, and triglycerides are the major components of milk fat. Since *GPAT4* gene is involved in glycerolipid biosynthesis and milk fat production in the mammary gland of cows,^6^^,^^9^ it has a significant influence on its quality and quantity.^3^^,^^7^ Based on the regulatory role of the *GPAT4* gene on milk composition,^12–14^ its single-nucleotide polymorphisms can serve as molecular markers for early animal evaluation to develop a marker-assisted selection strategy for increasing milk fatness in the cattle population.^15^

Owing to alternate exon-intron structure of protein-coding genes, their primary transcripts go through splicing process for introns removal from precursor messenger RNA (mRNA) and transformation into a mature mRNA, consisting of exons only.^16^ This splicing process occurs either during or immediately after transcription. The regulation of transcription and subsequent gene splicing are crucial to correct gene expression. However, in nature the exons of primary transcripts (pre-mRNAs) from genes can be spliced in different arrangements due to the presence of an alternative splicing^17^ during which multiple distinct functional and not functional transcripts are produced from a single gene. Various isoforms of mature mRNA, produced by many genes of higher eukaryotes, afterwards can be translated into numerous proteins. Alternative splicing is a key element in eukaryotic gene expression that increases the coding capacity of the cattle genome, but the selection of incorrect splice sites causes animal disease. Bioinformatic analysis shows that at least 70% of the alternative (non-principal) variants of human splice isoforms would lose important functional or structural information relative to the principal isoform.^18^

Since 95% of human genes and over 70% of genes in some plants are alternatively spliced, they considerably increase their proteome complexity^19^^,^^20^ thus showing an important mechanism for controlling gene expression in a cell/tissue-dependent, stage-dependent or stimuli- dependent manner.^16^^,^^21^^,^^22^ Bovine genes (35,092 in total; www.ncbi.nlm.nih.gov/datasets/taxonomy/9913/) show fewer alternative splicing events compared human (59,444 in total, www.ncbi.nlm.nih.gov/datasets/taxonomy/9606/) and mouse genes (49,244 in total; www.informatics.jax.org). Out of all the bovine genes, 4567 genes (13%) are alternatively spliced, compared with 16,715 (28%) in humans and 16,491 (33%) in mice.^23–25^

The specific role of alternative splicing in determining phenotypic variation in economic traits is not well understood, partly because of the lack of a comprehensive annotation of alternative splicing in agricultural species.^26^ There are on average more than a three-fold difference between annotated splice isoforms per human gene − 5.1, versus per cattle gene − 1.6, (5.1/1.6 ≈ 3.2).^27^

Alternative splicing patterns are generally assessed by transcript data examination.^28^ For any gene of interest, alternative splicing events can be identified by using reverse transcription polymerase chain reaction (RT-PCR) and a complementary DNA (cDNA) library. However, here we are applying the minigene approach based on the cell culture model in order to demonstrate, that specific nucleotide changes affect splicing efficiency^29^ in the *GPAT4* gene. The splicing outcomes may have different phenotypic consequences and are often challenging to predict without analysis of RNA samples from affected animals. Prediction results are not unambiguous. By creating two *GPAT4* minigens and sequencing analysis, we are able to evaluate the consequence of splice mutation in the *GPAT4* gene.

Thus, the aim of this study was: first, to identify polymorphisms of the *GPAT4* gene associated with milk fat synthesis in cattle and most likely leading to alternative splicing. Second, to confirm the viability of the obtained isoforms using functional minigene analysis.

## Materials and methods

Peripheral blood samples were collected in the middle of March of 2018 from the jugular vein of black-and-white Holstein adult clinically healthy cows of one dairy herd from the Kaluga region of Russia (54°36′ N u 36°00′ E). A veterinarian sampled the animals in accordance with the Institutional Review Board Statement and Informed Consent Statement (see below). Genomic DNA was isolated from 100 µl ethylenediaminetetraacetic acid disodium salt (EDTA-Na2) – preserved whole blood samples using M-Sorb Kit (Synthol, Moscow, Russia) according to the manufacturer’s instructions. DNA samples were analyzed spectrophotometrically (BioPhotometer Plus, Eppendorf, Germany) for quality and concentration determination and stored at −20 °C until used as a template for PCR.

The cattle reference genome for the Hereford Bos taurus assembly (ARS-UCD1.2; NC_037354.1 (2018/04/11)) was retrieved from the NCBI website.^30^ Searching for mutations in cattle’s gene *GPAT4* and their analysis was conducted with the use of database Ensemble,^31^ European Variation Archive (EVA),^32^ program HSF v. 3.1 (Human Splicing Finder),^33^^,^^34^ NNSplice^35^ and ASlive: a first database of its kind in livestock animals.^26^ The minigene construction

For functional analysis of the influence of single-nucleotide replacement in intron 2 of the *GPAT4* gene (rs442541537, 27:36531205 G/A) on splicing pattern used the minigene expression system *in vitro*. One pair of primers AGPAT6_F_XhoI atggggtagggatcaccagaattctggagctcgagttcttggaataacttgggaggtag and AGPAT6_R_XhoI accagatatctgggatcctgcagcggccgctcgagcagcaggaagtagggtccaa were designed to partially cover intron 1, exon 2, intron 2, exon 3 and partially intron 3 of the cattle *GPAT4* gene (the expected size of the PCR product is 1316 bp; annealing temperature Tm = 64 °C) using the NCBI primer BLAST online software. Primers were synthesized by Evrogen (Moscow, Russia).

The minigene of *GPAT4* was obtained by PCR amplification of exons 2, 3 with the corresponding introns on the template genomic DNA. The PCR reactions were performed in a 50 pl reaction mixture consisting 200 pM of each dNTPs (Thermo Fisher Scientific, Waltham, MA, USA), 0.5 pM of each primer, 1 × Q5 Reaction buffer and 0.01 U/pl Q5 Hot Start High Fidelity DNA Polymerase (New England Biolabs, Massachusetts, USA) and 160 ng of purified DNA sample as a template. The reactions were performed with an automated DNA thermal cycler (BioRad Laboratories, Inc., T100, CA, USA) with a 30 s denaturation step at 98 °C followed by 35 cycles. Each cycle consisted of a denaturing step of 10 s at 98 °C, an annealing step of 30 s at 64 °C, and an elongation step of 90 s at 72 °C and a final extension at 72 °C for 2 min.

Amplification products from positive samples were analyzed by agarose gel electrophoresis and expected bands were excised and purified by the Cleanup Mini Gel DNA Recovery Kit (Evrogen, Moscow, Russia) according to the manufacturer’s instructions. The PCR product (1316 bp) was cloned into pSpl3-Flu2 vector using XhoI restriction sites after digestion by Sfr274I (Sibenzyme, Russia) (isoshisomer of XhoI) according to instructions. A recombinant plasmid vector was used for competent cells *E. coli* Top10 transformation according to the manufacturer recommendations. For PCR colony screening, the following primers were used for minigene insert detection pSPL3-f GGAGAAATATCAGCACTTGTGGA and pSpl3-Flu2R TAATTTCTCTGTCCCACTCCA. After plasmid DNA purification by Plasmid Miniprep (Evrogen, Moscow, Russia), three positive clones from each sample were sequenced bidirectionally by the Sanger method at Evrogen JSC facilities (Moscow, Russia).

For single-site mutation two PCR mix reactions of 25 pl each were prepared with a pair of two 39 bp primers (FOW and REV), where 30 bp of both have to overlap and mutation located 18 bp from 5′ site of the primer. Primers were synthetized by Evrogen (Moscow, Russia). Mix1 (final concentration is shown): 2.5–10 ng of DNA template (plasmid Miniprep dilued 1/10); 0.25 pM of primer FOW; 200 pM of dNTPs; 5 pl 5x HF buffer; 1 U Phusion Hot start II High-Fidelity DNA polymerase; 2.5 U taq polymerase. Mix2: 2.5–10 ng of DNA template (plasmid Miniprep diluted 1/10); 0.25 pM of primer REV; 200 pM of dNTPs; 5 pl 5x HF buffer; 1 U Phusion Hot start II High-Fidelity DNA polymerase; 2.5 U taq polymerase. The PCR cycles for both Mix1 and Mix2 were initiated at 95 °C for 30 s to denature the template DNA, followed by 3 amplification cycles. Each amplification cycle consisted of 95 °C for 30 s, 55 °C for 1 min and 68 °C for 6 min. The PCR finished with 14 °C for 10 min. After insertion Mix1 and Mix2 in the same PCR tube and denaturation step for the mixture at 95 °C for 30 s, 22 cycles of the following sequence was applied: 95 °C for 30 s, 55 °C for 1 minute, 68 °C for 6 min. The PCR was finished with 14 °C for 10 min. The resulting PCR product was treated with Dpnl enzyme: 15 µl of PCR product + 1 µl of Dpnl (20 U) was incubated at least 2h 30 min (or until overnight incubation) at 37 °C. The digested product was transformed into *E.coli* Top10 competent cells by heat shock at 42 °C for 90 s and some clones were sequenced to verify the mutation after overnight incubation at 37 °C and the plasmid DNA isolation. DNA sequencing was performed at least four times on both sides using the Evrogen Sequencing Service (Evrogen, Russia). The PCR cycles, DNA quantification, transformation and mutation verification were essentially the same as described above.

Transplanted human embryonic kidneys HEK293T cell line (obtained from the Bank of the Institute of Innovative Biotechnologies in Livestock, Moscow, Russia) were cultured at 37 °C in an atmosphere of 5% CO_2_, in DMEM/F12 medium (PanEco, Russia) in a 1:1 ratio with the addition of 10% fetal calf serum (PanEco, Russia), gentamicin at a concentration of 0.09 mg/ml, 2.5 p.g/ml amphotericin B and 2 mM glutamine.

The cells were removed from the wall of growing flasks with 0.05% trypsin-EDTA solution (PanEco, Russia) and planted in a 24-well culture plate at a concentration of 6.0 × 104 24 hours later, the plasmid pSpl3-Flu2 with insertion of GPAT4 minigene was used for transient transfection according to commercial protocols. Before this, the following transfection reagents Lipofectamin 2000 (Thermofisher, USA), Lipofectamin 3000 (Thermofisher, USA), and Turbofect (Thermofisher, USA) were tested for plasmid pSpl3-Flu2 transfection.

Live cells were analyzed by fluorescence microscopy and images were captured using an inverted Axio Observer 7 system (Carl Zeiss) with objective PlanApochromat 63 × 1.46 Oil 24–48 h after transient transfection. The following filter cube was used: green fluorescent protein (GFP) (excitation filter 450–490 nm, a beam splitter 495 nm, emission 500–550 nm) and Katushka (excitation filter 540–580 nm, a beam splitter 585 nm, emission 593–668 nm). The image processing and analysis was carried out with use of an open source software ImageJ (distributed without license restrictions as the public domain). Far-red fluorescent protein Katushka and GFP, provide bright and spectrally non-overlapping signals.

The cells grown in a monolayer were lysed with ExtractRNA reagent (BC032, Evrogen, Moscow, Russia) solution at room temperature and homogenized. Total RNA was extracted according to the commercial instruction (supplied for ExtractRNA reagent BC032). Briefly, the method is as follows. After lysate centrifugation (12,000–15,000 g, 10 min) and removal of the sediment and fat film, 0.2 ml of chloroform was added for every 1 ml of ExtractRNA reagent. The mixture was vigorously stirred for 15 s and incubated for 3–5 min at room temperature (RT), with occasional shaking. After centrifugation (12,000 g, 15 min at 4 °C) and separation of the mixture into three phases, the upper colorless aqueous phase containing RNA was selected. 0.5 ml of 100% isopropanol was added to the aqueous phase (for every 1 ml of ExtractRNA reagent) and the mixture was incubated at RT (10 min). After sample centrifugation (12,000 g, 10 min at RT) and supernatant collection, the desired RNA precipitate remained at the bottom of the tube. After adding 2 ml of 75% ethanol for every 1 ml of isopropanol used, the sample was centrifuged at maximum speed (5 min at RT). The ethanol was removed and the precipitate was drying (5–7 min). The total RNA was dissolved in the required volume of RNAse free water for immediate use, or stored in aliquots at −80 °C. Reaction of reverse transcription carry out with use of Mint reverse transcription system (Evrogen, Moscow, Russia). The synthesis of DNA from prepared an RNA template, via reverse transcription, results in complementary DNA. For study of minigene isoforms conduct cDNA amplification with plasmid specific primers TurboFP-F ACAAAGAGACCTACGTCGAGCA and GFP-R AGCTCGATCAGCACGGGCACGAT. Amplification products were analyzed by agarose gel electrophoresis and expected bands were excised and purified by the Cleanup Mini Gel DNA Recovery Kit (Evrogen, Moscow, Russia) according to the manufacturer’s instructions and Sanger DNA sequencing at least four times on both sides was carried out using the Sequencing Service (Evrogen, Moscow, Russia) with primers TurboFP-F and GFP-R.

## Results

The Ensembl genome database has related thirteen mutations to the splicing events in the *GPAT4* gene. A single nucleotide substitution have been selected directly next to the location of the exon in the *GPAT4* gene, that changes the first base region within the non-coding site in a gene at the 5′ end of the second intron rs442541537, 27:g.36531205 G > A. Because of splice site prediction software anticipate this SNP as a highly confident splice donor variant site in a conserved position,^1^^,^^33^^,^^35^^,^^36^ then one would expect it to have an effect on splicing. Moreover, it would be expected that mutation in the splice-donor sequence may lead to disruption of spliceosome binding contact and consequently to alternative splicing event. Accordingly, the selected SNP is highly likely to produce mature mRNA with different functionality, the impact of which must be assessed and confirmed.

To determine the influence of the investigated mutation on the splicing of pre-mRNA used the model system of minigene expression in the cell line. For the vectors creation containing a fragment of target *GPAT4* gene (minigene) wild type (WT), we amplified the part of genomic DNA of the studying sample consisting of an alternative second exon of interest flanked by adjacent upstream and downstream introns and third exon of *GPAT4* gene with a third subsequent intron. All this construction is cloned between Katushka and GFP-encoding sequences in the vector of pSpl3- Flu2. The missing mutated variant with nucleotide A of mutant (MUT) instead of G of WT was previously synthesized based on the wild type (rs442541537, 27:36531205 G > A). The identification of single-site mutation for WT and MUT was completed by sequencing. Each of the two constructions was transfected into the cell line Human Embryonic Kidney (HEK) 293. Because the plasmid contains two fluorescent proteins: Katuschka (red) and GFP (green), yellow color is the result of the joint fluorescence from two luminous proteins expressed in cells. Cells expressing these constructs develop different levels of red and green fluorescence, and the ratio of red to green can be used to estimate a proportion of normal and alternative transcripts.

**Figure 1.**
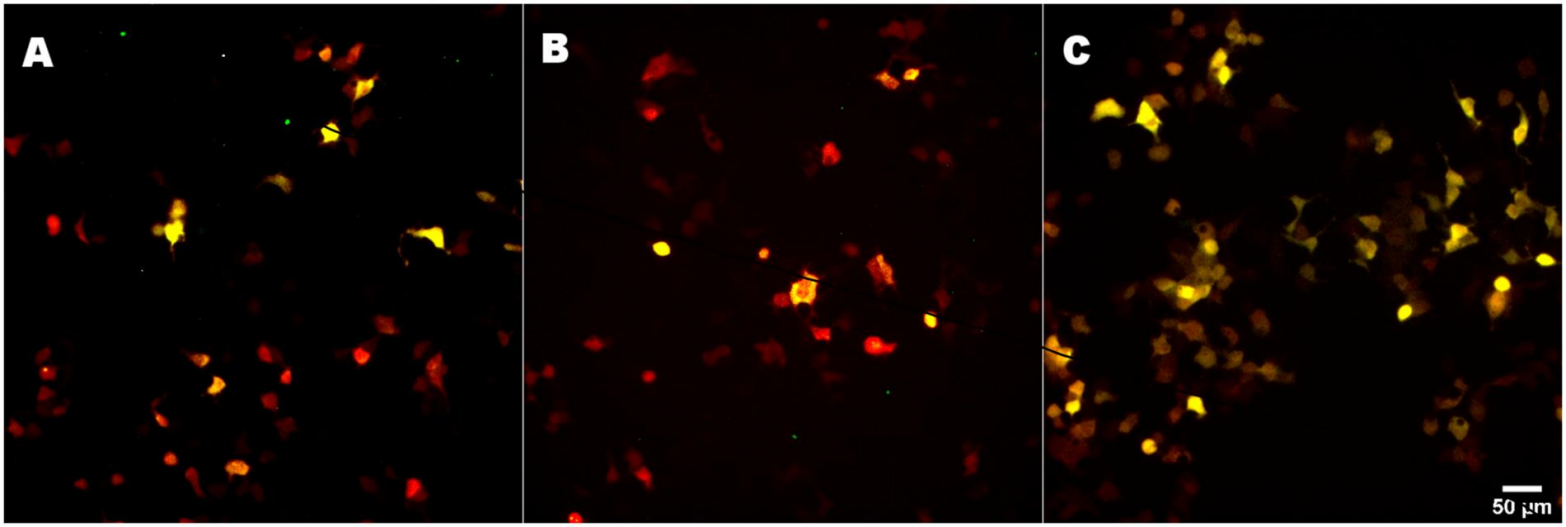
Transfection of HEK293T cells with the plasmid pSpl3-Flu2 and different transfection agents: (A) lipofectamine 2000, (B) lipofectamine 3000 and (C) turbofect.

Transfection effectiveness determination was carried out with a pSpl3-Flu2 vector in a model cell line used with three different transfection agents. The highest transfection efficiency about 85% was attained by the use of the turbofect agent (Fig. 1C). At the same time, agents lipofectamine 2000 and lipofectamine 3000 both have similar efficiency only ∼ 40% (Fig. 1A,B).

**Figure 2.**
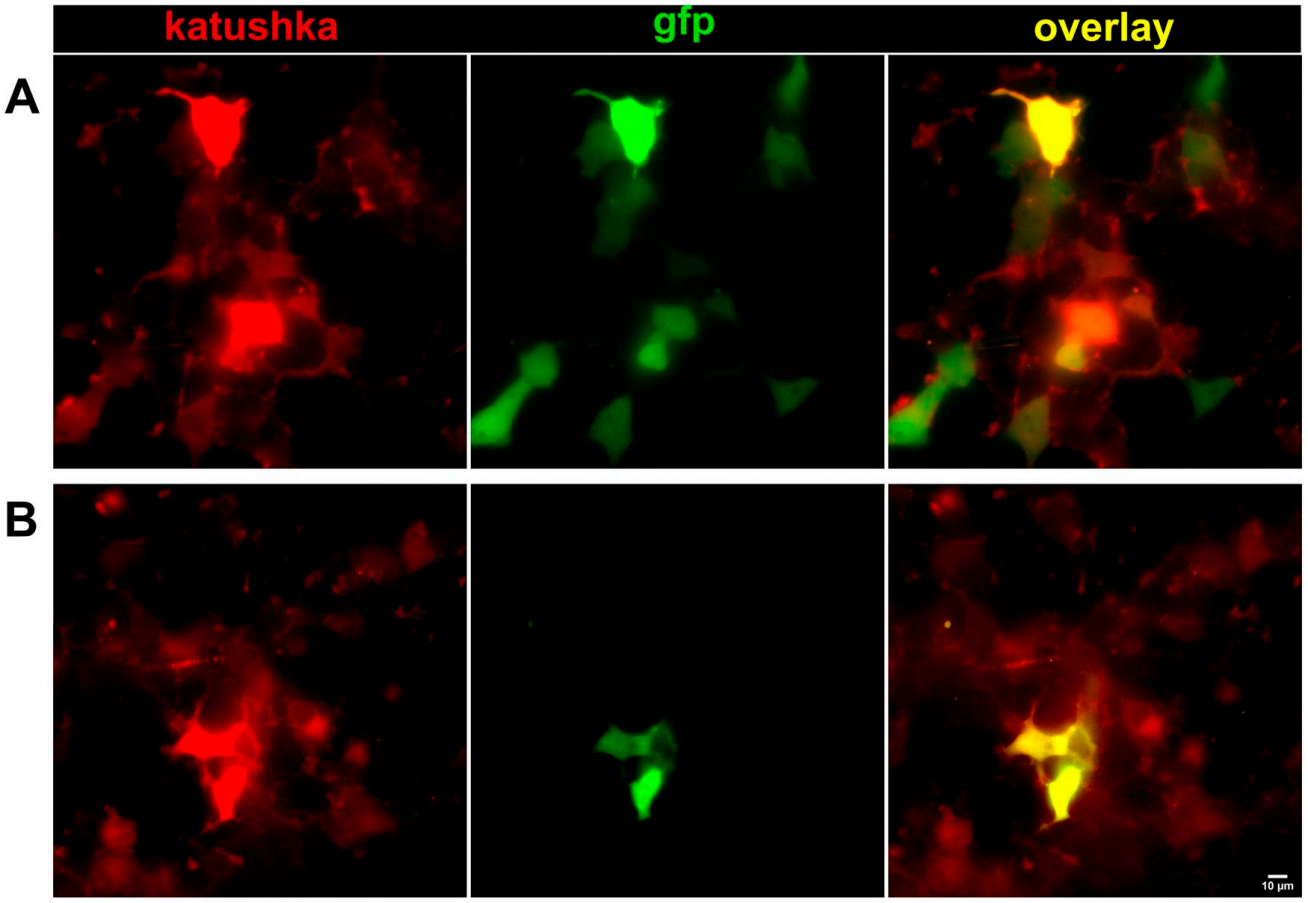
Fluorescence microscopy of HEK293T cells transiently expressing target vectors. (A) WT; (B) MUT.

The differences in colors of two fluorescent proteins expressed together with minigene clearly show a distinction between splicing variants taking place in MUT and WT (Fig. 2A,B). Translation of the normally spliced full-length transcript results in a fusion protein, containing both fluorescent proteins Katushka and GFP (Fig. 2A).

In contrast, alternative second exon skipping results in a frame shift within the third exon and thus leads to a truncated protein without GFP (Fig. 2B). In the experimental construct undergoing alternative splicing, an additional red signal, which is above the estimated control red/green ratio, corresponds to the alternative transcript.

**Figure 3.**
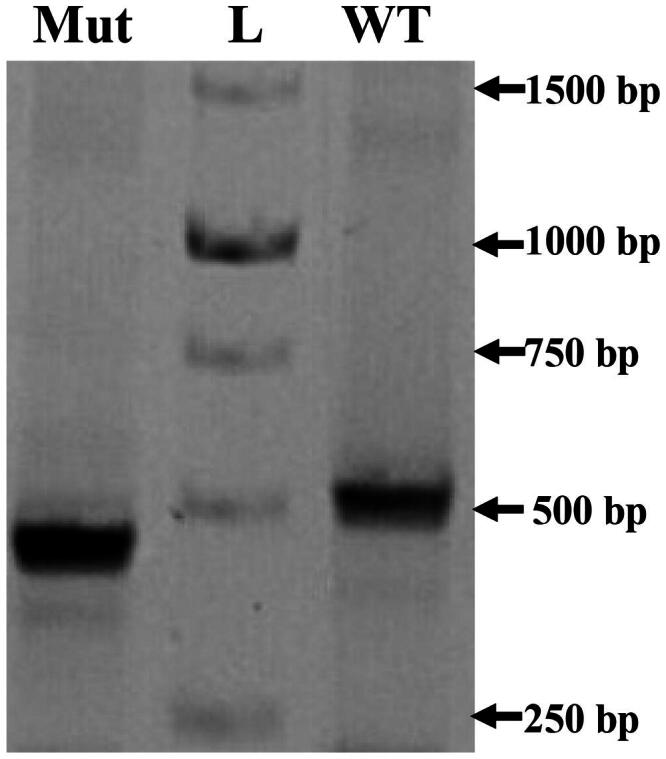
Fragment of agarose gel electrophoresis of the PCR amplification (primers TurboFP-F and GFP-R) products pSpl3-Flu2-WT and pSpl3-Flu2-MUT with one nucleotide replacement of cDNA sequences generated by transfected HEK293T cells. Lane L = marker DNA 1 kB DNA ladder (1500, 1000, 750, 500, and 250 bp) (Evrogen, Moscow, Russia).

**Figure 4.**
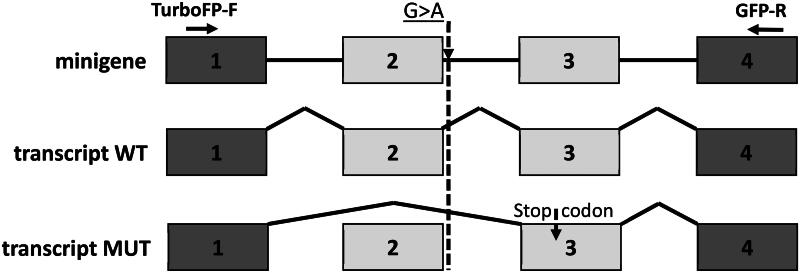
The fragment of two exon trapping vector pSPL3-Flu2 used to assay SNP function. Schema of WT and MUT pSPL3-Flu2 plasmids containing 399 bp of intron 1, 70 bp of exon 2, 130 bp of intron 2, with studied splice donor site nucleotide substitution, 302 bp of exon 3 and 361 bp of intron 3 were separately cloned into the XhoI cloning sites of the pSPL3-Flu2 vector and plasmid specific primers for cDNA amplification. Exons of mRNA *GPAT4* are numbered according to NC_037354.1.

Forty-eight hours after transfection total RNA was extracted from these cells and reverse transcription was applied. The resulting cDNA was amplified with the use of plasmid specific primers. Because of splicing, we found the difference in mobility of two PCR products, received from minigene with mutation against wild type (Fig. 3). Sanger sequencing of these fragments show that in the presence of mutation rs442541537, 27:36531205 G > A (MUT) in the second intron, the whole second exon is omitted from the spliced transcript. Consequently, studied SNP mutation of the second intron lead to a switch off splicing site and this result in a second exon deletion (Fig. 4) and a frame shift in exon three. Thus, expression of *GPAT4* minigene produces two splice variants: full-length transcript consisting of all four exons (WT) and minor short transcript lacking a second exon of 70-bp length (MUT).

Therefore, we have identified the alternatively spliced transcript isoform that had lost the second exon, altered the reading frame and introduced a stop codon in the adjacent downstream 3-d exon of RNA of *GPAT4* gene from cow.

## Discussion

In the present study, we have characterized a single splice site mutation in the *GPAT4* gene. We have shown that mutation of the canonical splice site of the *GPAT4* gene causes a splicing error and leads to several splice-site uncoupling events.^37^ Two of these were the most significant. The first was the absence of the entire second exon of seventy nucleotides in length in the spliced transcript in the mature mRNA. The second manifested itself in the appearance of a stop codon in the third exon, resulting in a truncated, nonfunctional protein. As indicated earlier, exon skipping and aberrant activation of splicing sites account for the majority of splicing site mutations in higher eukaryotes.^38^ Most pathological splice site mutations are single-nucleotide substitutions located either in canonical GT splice donor sites (+1/+2) or in AG acceptor sites (−1/−2).^39^

Numerous databases and online bioinformatic tools are available to detect and analyze alternative splicing. Their replenishment for mutations localized outside the coding sequences and causing pathogenic mutations is rather slow and does not fully reflect their extensive influence on the impairment of splicing.^40^ Recently, an animal database ASlive (ASlive.org) specifically targeting alternative splicing was created.^26^ However, these programs and databases do not offer direct prediction of the functional consequences of splice-site mutations leading to various metabolic abnormalities.^41^ The Ensemble database mentions about 13 intronic single nucleotide polymorphisms associated with *GPAT4* splicing, including the SNP G/A (rs442541537; 27:36531205). The automated database annotation does not provide references or specific data on sequence variations or consequences for the transcript produced by the mutation. Accordingly, we made our own prediction based on probabilistic estimates and analyzed RNA samples from animals with such changes in the functional assay. In the absence of a mutant variant, cloning and expression of the defective gene was performed for RNA analysis. The entire *GPAT4* gene is too large to be cloned and expressed for splice mutation studies (https://www.ensembl.org). By sequencing two *GPAT4* minigens (native and mutant variants) by site-directed mutagenesis and functional analysis in a cell model system with appropriate control sequencing, we were able to assess the consequences of splice mutations, which could not be fully predicted by existing programs.

Identification of actual splicing sites and correct deletion of introns are necessary to obtain the desired mRNA isoforms and the proteins they encode.^42^ It was shown that the MUT variant, unlike WT, produces an isoform without the second exon of *GPAT4*. Alternatively, spliced transcript isoforms that lost the second exon changed the reading frame and inserted a stop codon into the neighboring downstream 3-d exon of the cow *GPAT4* gene RNA. Since all the important conserved functional motifs of the GPAT4 acyltransferase are located further than the stop codon in the sequence,^11^ it is conceivable that this could lead to the production of a nonfunctional protein. Since in our case excision of the second exon from the final gene transcript resulted in a shortened mRNA variant, we were dealing with the best known mechanism of alternative gene splicing in mammalian pre-mRNA, called exon skipping (or cassette exon).^43^^,^^44^ According to some estimates, this type of splicing accounts for more than 38% of alternative splicing events in mammals.^45^

Alternative splicing of the target cassette exon in the *GPAT4* fragment (minigene) inserted into the pSPL3-flu2 plasmid was analyzed using two fluorescent proteins Katushka + GFP, of different colors, red and green, respectively. The transcription of the red fluorescent protein occurred in both normal full-length and alternative (exon-skipping) forms. The red fluorescent signal corresponds in this case to the alternative (Katushka) and is mixed with normal (Katushka + GFP) transcripts in the control sample (Katushka + GFP). Thus, the arrangement of fluorescent proteins allowed us to visualize the result of splice site mutations using UV illumination immediately after transfection.^46^

It has been previously described that the five most significant variants in the *AGPAT6* gene are located in the upstream region (at 36,209,319; 36,211,252; 36,211,258; and 36,211,708 bp) or in the 5′-UTR regions (at 36,212,352 bp) of the *AGPAT6* gene.^47^^,^^48^ Daetwyler et al. identified four variants located in the upstream region as candidate polymorphisms for the cause of the disease in both Holstein and Fleckvieh cows.^49^ The authors indicated that the indel polymorphism at 36,211,252 bp was the most likely causative mutation because it was highly likely to be located within the transcription factor binding site. At the same time, Littlejohn et al.^48^ described strong associations between cow milk composition traits (fat, protein, and lactose) and 10 variants in the *AGPAT6* gene in the 5′UTR regions of exons 1 and 2 as well as the connecting sequence of intron 1. The 36,212,352 bp variant appears to be the most likely causative mutation because of its location in the 5‘UTR region of the AGPAT6 gene. Four *AGPAT6* polymorphisms (NC_007328.3:g.152G > C, 8124 G > A, 9263 C > G, and 16436 G > A) were also found in the 5‘UTR, intron 2, exon 4, and 3‘UTR, respectively, in dairy goats,^13^ but the mechanism of the *AGPAT6* intronic mutation (NC_007328.3:g. 8124 G > A) has not been explained.

Mutations in *GPAT4*, which encodes the enzyme glycerol-3-phosphate acyltransferase, are associated with various activities and may be a potential therapeutic target for the prevention and treatment of diseases related to energy metabolism,^14^ obesity, insulin resistance, and type 2 diabetes.^47^

Moreover, *GPAT4* is a functional gene responsible for milk fat content with pleiotropic effects on other milk components, particularly protein content.^48^ For this reason, considerable attention has been paid to the single nucleotide polymorphism of this gene in dairy farming,^50^ where an association between genetic polymorphism and mammalian milk quality has been established.^51^^,^^52^ Alternative splicing is a major mechanism of functional regulation, reaching one-fifth of the genes subjected to it in cattle.^23^ Therefore, functional mutations responsible for phenotypic traits are the most efficient choice of markers to detect genetic defects and, consequently, to predict the genetic potential of animal productivity immediately after birth.^53^

## Conclusion

Thus, we performed a functional analysis of a single splice-site mutation of the *GPAT4* gene using a specialized minigene splicing assay, which offers a simple and reliable method to analyze the results of splice-site mutations. The process of validation of the *GPAT4* gene polymorphism led to the discovery of a single nucleotide responsible for the manifestation of alternative splicing. As it was assumed, the sequence variation studied guided to several events resulting from the splicing site disconnection. The most significant is the absence of the entire second exon in the spliced transcript in the mature mRNA and the appearance of a stop codon in the third exon, resulting in the occurrence of a truncated nonfunctional protein. Validation of the studied mutation as pathogenic will be important for replenishment of databases of mutations localized outside the coding sequence and affecting splicing disruption. The biological significance of alternative splicing of *GPAT4* and its association with milk fat production in cows remains to be explored. However, pathogenic splicing variants have become an important target of genomic veterinary medicine, and the studied sequence variation affecting splicing can already be used for the prevention of metabolic disorders. Therefore, we consider the further development of this work to be validation of the studied mutation as pathogenic, which will be important for enrichment of databases of mutations localized outside the coding sequence and affecting the splicing disorder. The studied SNP may be useful for developing an understanding of the functional importance of the *GPAT4* as a candidate gene for milk fat synthesis and, consequently, becoming a genetic marker for breeding strategies in dairy cows.
